# Use of Wastewater to Monitor Antimicrobial Resistance Trends in Communities and Implications for Wastewater-Based Epidemiology: A Review of the Recent Literature

**DOI:** 10.3390/microorganisms13092073

**Published:** 2025-09-05

**Authors:** Hannah B. Malcom, Devin A. Bowes

**Affiliations:** Department of Environmental Health Sciences, Arnold School of Public Health, University of South Carolina, 921 Assembly Street, Suite 401, Columbia, SC 29208, USA; hmalcom@email.sc.edu

**Keywords:** wastewater, antimicrobial resistance, resistance genes, antibiotics, wastewater-based epidemiology, public health, framework

## Abstract

Antimicrobial resistance (AMR) presents a global health challenge, necessitating comprehensive surveillance and intervention strategies. Wastewater-based epidemiology (WBE) is a promising tool that can be utilized for AMR monitoring by offering population-level insights into microbial dynamics and resistance gene dissemination in communities. This review (*n* = 29 papers) examines the current landscape of utilizing WBE for AMR surveillance with a focus on methodologies, findings, and gaps in understanding. Reported methods from the reviewed literature included culture-based, PCR-based, whole genome sequencing, mass spectrometry, bioinformatics/metagenomics, and antimicrobial susceptibility testing to identify and measure antibiotic-resistant bacteria and antimicrobial resistance genes (ARGs) in wastewater, as well as liquid chromatography-tandem mass spectrometry to measure antibiotic residues. Results indicate *Escherichia coli*, *Enterococcus* spp., and *Pseudomonas* spp. are the most prevalent antibiotic-resistant bacterial species with hospital effluent demonstrating higher abundances of clinically relevant resistance genes including *bla*, *bcr*, *qnrS*, *mcr*, *sul1*, *erm*, and *tet* genes compared to measurements from local treatment plants. The most reported antibiotics in influent wastewater across studies analyzed include azithromycin, ciprofloxacin, clindamycin, and clarithromycin. The influence of seasonal variation on the ARG profiles of communities differed amongst studies indicating additional factors hold significance when examining the conference of AMR within communities. Despite these findings, knowledge gaps remain, including longitudinal studies in multiple and diverse geographical regions and understanding co-resistance mechanisms in relation to the complexities of population contributors to AMR. This review underscores the urgent need for collaborative and interdisciplinary efforts to safeguard public health and preserve antimicrobial efficacy. Further investigation on the use of WBE to understand these unique population-level drivers of AMR is advised in a proposed framework to inform best practice approaches moving forward.

## 1. Introduction

Antimicrobial resistance (AMR) has been increasing in severity over the last decade, contributing to nearly 5 million deaths in 2019. In the U.S., over 2.8 million cases of antimicrobial resistance-related infections occur each year, with an estimated 1.27 million deaths directly attributable to AMR worldwide [1]. Concerns involving antibiotic-resistant bacteria began nearly a century ago as infections grew unresponsive to treatment, particularly for *Staphylococcus* spp. In 1976, the World Health Organization (WHO) developed working groups dedicated to antibiotic resistance, and in 1994, declared antibiotic resistance as a major public health problem, influencing the creation of the 2001 Global Strategy for Containment of Antimicrobial Resistance [2]. Today, six types of bacterial antibiotic-resistant hospital infections (carbapenem-resistant Enterobacterales (CRE), carbapenem-resistant *Acinetobacter*, Methicillin-resistant *Staphylococcus aureus* (MRSA), Vancomycin-resistant *Enterococcus* (VRE), extended-spectrum beta-lactamase (ESBL)-producing Enterobacterales and multidrug-resistant (MDR) *Pseudomonas aeruginosa*) have increased by 20% as a result from the COVID-19 pandemic, which have remained above pre-pandemic levels as of 2022 [3]. Additionally, reported clinical cases of antifungal resistant *Candida auris* have increased nearly five-fold since 2019, raising concerns about the viability of current treatment options, and what may lie ahead in the future [3].

Given the complexity of AMR, multidisciplinary and dynamic approaches are required to effectively monitor and assess the status and progression. Thus, to survey global AMR, the WHO enacted the Global Antimicrobial Resistance and Use Surveillance System (GLASS) which is focused on collecting epidemiological, clinical, and population-level data grouped into a series of technical modules that provide readily available data from clinical trials, antimicrobial consumption, and point prevalence surveys on antibiotic use in hospitals. However, while the data obtained from GLASS efforts are informative, there are several challenges that accompany this effort, such as disproportionate access to data across the globe, particularly impacting low-middle income countries (LMICs), which hinders progress on assessing global monitoring and tracking of AMR. Additionally, data collection challenges such as misclassification and underreporting further complicate the ability to fully assess the current status of the situation [4]. Overall, these can lead to gaps in knowledge that are pertinent to understanding the breadth of the problem from a comprehensive and data-driven perspective.

A promising approach for acquiring rapid, minimally invasive, objective, and comprehensive data on AMR in communities is wastewater-based epidemiology (WBE) where human excreted biomarkers indicative of activity, behavior, health, and exposure are analyzed in untreated community wastewater [5]. Impacts from climate change and the use of harmful chemicals that contaminate our environment continue to pose a threat to the health of human populations. The field of WBE has evolved significantly in recent years due to its widespread use during the COVID-19 global pandemic; however, investigating the utility of this application to fit within a broader environmental public health framework that focuses on antimicrobial resistance remains unexplored. Now, in the post-pandemic era and leveraging an established global network of experts, the use of WBE can be applied to complement current strategies to support rapid surveillance of AMR in communities [6]. Currently, major gaps in WBE for AMR exist, including a lack of standardized sampling, normalization, and analytical methods, which limit comparability across studies. There is also a persistent disconnect between ARG detection and clinically relevant resistance, with limited studies investigating the use of wastewater to monitor AMR and assess public health outcomes. Additionally, long-term, multi-site datasets and integration with One Health surveillance remain scarce, hindering WBE’s transition from promising research to actionable monitoring.

Thus, given the severity of antimicrobial resistance, the importance for public health significance, and the identified utility of WBE to monitor AMR in communities in near real-time, we conducted a scoping review of the recent literature with the following objectives: (1) understand the current status of using WBE for AMR surveillance in populations, (2) identify pertinent gaps in the existing literature, and (3) provide recommendations to be addressed in future work.

## 2. Methods

### 2.1. Literature Search

Over the course of half a decade, the field of WBE has significantly evolved due to the COVID-19 pandemic, allowing for expansion into other areas such as AMR. Thus, we examined the recently published literature between January 2019 and April 2024 using the PubMed (National Library of Medicine) and ScienceDirect databases to assess the current status of analyzing influent wastewater for AMR to gauge public health status and inform surveillance strategies. The following keywords were used to search for published works: (antibiotic resistance genes OR antimicrobial resistance) AND (wastewater-based epidemiology OR wastewater OR sludge OR influent). The initial search yielded 2611 results on PubMed and 10,353 results on ScienceDirect.

### 2.2. Inclusion and Exclusion Criteria

**Figure 1:**
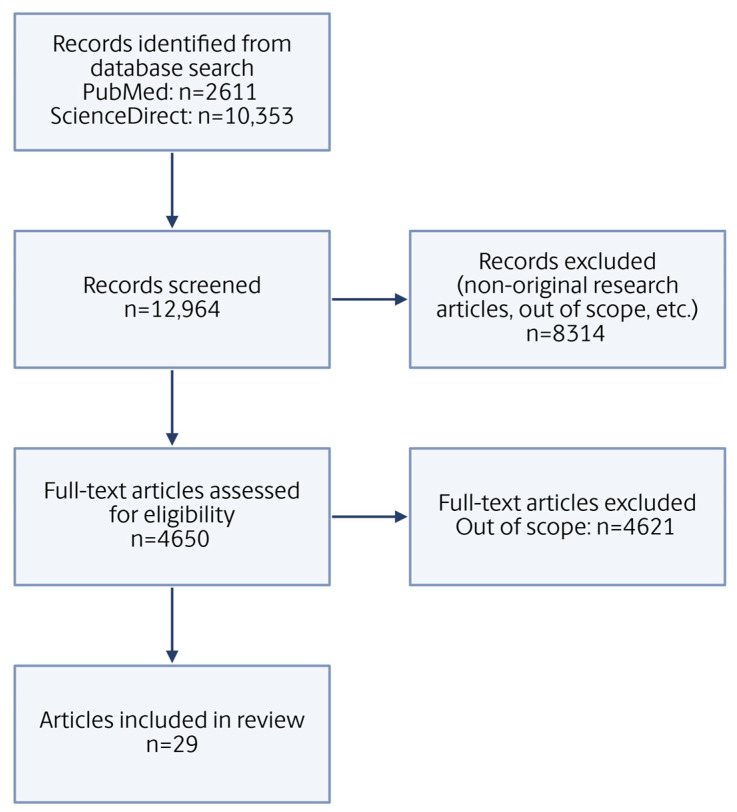
Schematic of the literature search process based on PRISMA guidelines.

The initial inclusion process involved any published works that populated during the keyword search. Next, a title and abstract screening was conducted to exclude any papers that were not original research (i.e., reviews, meta-analysis, etc.), resulting in excluding 8314 papers. Any duplicates were identified and removed. Other papers that did not fit within these criteria (e.g., focus on wastewater treatment and/or post-treatment, etc.) were considered out of scope for the purpose of this review, and subsequently excluded (4621 papers). After all the aforementioned conditions were excluded, the search yielded 29 original research papers deemed within scope for the purpose of this scoping review (Figure 1).

### 2.3. Data Extraction and Analysis

All twenty-nine articles included for this study based on the criteria above underwent rigorous analysis and were evaluated and categorized based on the following criteria: overall methodology, laboratory methods and protocols, results, limitations, and final overall conclusions and suggestions for future work. Primary points of interest included the analysis of wastewater influent (and/or sludge in some cases), quantitative data for measured antibiotics, ARGs, and/or antimicrobial resistant bacterial species in those specific matrices and framed their findings in the context of public health. These papers were also examined to identify similarities or differences amongst the work to identify gaps in the current literature and inform recommendations for future work.

## 3. Results and Discussion

**Figure 2:**
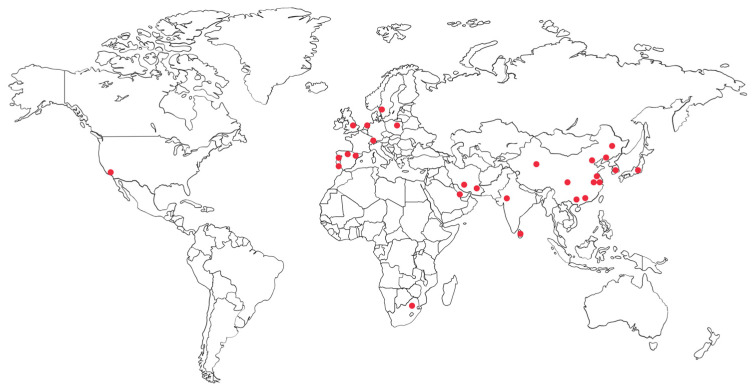
Map of collection sites showcasing global sample collection distribution of the papers analyzed in this review. Each red dot represents a reported wastewater collection site.

We reviewed and analyzed the recent literature (29 papers, <5 years) to investigate the current status of leveraging untreated wastewater as a valuable matrix to provide information on antimicrobial resistance development within communities across the globe (Figure 2) and identify actionable next steps to fill existing gaps in the scientific literature. Table 1 offers a summary of all studies analyzed based on sample collection approaches, laboratory methods utilized, results, and recommendations for future work (See Table S1 for comprehensive information).

### 3.1. Antibiotic-Resistant Bacterial Species Detected in Wastewater

Out of the literature reviewed, 15 articles (51%) investigated the bacterial community in influent wastewater (Table 1). Detection methods included conventional PCR [7,8,9,10], digital PCR [11], qPCR [12], metagenomics [13,14], whole-genome sequencing [15], antimicrobial susceptibility testing [8,15,16,17], bioinformatics [18,19,20,21], mass spectrometry [10], and culture-based [7,16,17]. Variation in detection methods exists across the reviewed literature for a variety of reasons including but not limited to funding, time constraints, and the scope of the paper.

Of those 15 papers, the most predominant phyla detected included Proteobacteria, Bacteroidetes, Actinobacteria, and Firmicutes [9,10,11,12,13,15,18,20]. The most common genera reported include *Escherichia*, *Pseudomonas*, *Aeromonas*, *Enterococcus*, and *Acidovorax* [7,8,9,14,15,16,17,18,19,20,21]. Of those genera, *Escherichia* and *Pseudomonas* emerge as two of the most commonly reported in wastewater that harbor antibiotic resistance genes [7]. Additionally, *Pseudomonas* spp. frequently emerges as one of the most dominant pathogens identified in raw wastewater [18,21], and *Enterococcus* has been identified as the most predominant culturable pathogenic multi-resistant bacteria (MRB) that exhibited extreme resistance to various antibiotics [15].

Other notable genera reported in the literature reviewed include *Klebsiella* spp., *Bacillus* spp., *Shigella* spp., and *Enterococcus* spp. [16,18]; all of which have human pathogenic capabilities such as urinary tract infections, wound infections, food poisoning, and systemic infections, and can carry and transmit antibiotic resistance genes [22]. Wastewater influent that included stormwater input in Switzerland was found to contain four out of five hosts of mobile genetic determinants (MGDs) were potential pathogens. These species included *Pseudomonas aeruginosa*, *Escherichia coli*, and *Citrobacter* spp. [23].

### 3.2. Antibiotic Resistance Genes Detected in Untreated Wastewater

Twenty-four studies (82%) detected and measured ARGs in wastewater influent predominately using qPCR or sequencing-based methods (Table 1). The primary genes identified and investigated for antibiotic resistance include *bla* genes, *sul1/2*, *ermB/F*, *intl1*, *qnrS, mcr/mbr*, and *tetM*. In one instance, bacitracin-associated ARGs (*mbr*) were shown to be the most predominant in rural influent wastewater (27.8%) which differed from municipal wastewater where beta-lactam-associated ARGs (*bla*) were the most predominant [20].

A study conducted in Japan showed that the 16S rDNA, *intl1*, and four target ARGS (*sul1/2* and *tetG/M*) were consistently detected in wastewater during the entire two-month sampling period from seven sites [24]. In northern China, *sul1* was found in all wastewater samples collected from five outlets within an ornamental fish market in October 2018 [9]. The ARGs *ermB/F*, *intI1*, and *sul1* were some of the genes selected for a study to identify candidate biomarkers of antibiotic resistance for wastewater monitoring because they have been previously identified as being a candidate for resistance due to their association with anthropogenic constituents in wastewater influent [25]. Results from this study suggest that when other targets are below the limit of detection, *intI1* may still be used to indicate the presence of low-abundance ARGs. Additionally, in a 13-month English study that measured ARGs in both community influent and hospital effluent, *intI1* was also identified as a potential marker of anthropogenic pollution due to its prior association with a diverse range of genes that confer resistance to a variety of substances such as antimicrobial agents (i.e., antibiotics, antivirals, antifungals, etc.), metals, and disinfectants [6].

Furthermore, the genes *sul1, intI1, and tnpA* specifically demonstrated potential to serve as genetic markers for antibiotic resistance risk within communities in a study that analyzed river water upstream and downstream from a WWTP as well as WWTP effluent during two sampling campaigns over the course of ten months [26]. Another study out of Beijing, China found a high correlation between antibiotics and ARGs such as in the case of sulfonamides being correlated with *sul1* (r = 0.787, *p* < 0.01). This study not only demonstrated this positive correlation with ARGs in sewage, but it showed a negative correlation between ARGs in sludge as a broad-spectrum fluoroquinolone (enrofloxacin) was negatively correlated with *tetW* (r = −0.887, *p* < 0.05), *ermB* (r = −0.933, *p* < 0.01), and *qnrS* (r = −0.938, *p* < 0.01). This suggests that optimal pharmaceutical removal in wastewater is vital for the reduction in ARG dissemination in downstream applications [27].

### 3.3. Antimicrobial Residues Measured in Untreated Wastewater

Seven out of the twenty-nine studies (24%) reviewed measured antibiotic residues in influent wastewater (Table 1). The predominant antibiotic classes reported across the literature analyzed are macrolides, beta-lactams, tetracyclines, fluoroquinolones, and bacitracin [20,23,28]. Results from the analyzed studies suggest that each of these classes has the potential to contribute to the selective pressure driving antibiotic resistance within community sewersheds. For example, it was shown that high antibiotic resistance rates in hospital wastewater effluent were found for sulfamethoxazole as *E. coli* and *P. aeruginosa* were able to grow at a concentration of up to 64 µ/L. The ratio of bacteria grown in sulfamethoxazole (32 µg/L) on EMB agar was 0.75, and on Cetrimide agar the ratio was 0.60 [7].

Of the most commonly detected antibiotics in wastewater and most consumed antibiotics without a prescription in Spain, 11 antibiotics (azithromycin, cefalexin, ciprofloxacin, clarithromycin, clindamycin, erythromycin, metronidazole, OH-metronidazole, sulfamethoxazole, N-acetyl-sulfamethoxazole, and trimethoprim) were measured in influent wastewater [29]. It was observed in this study that of 38 pharmaceuticals measured, antibiotics showed the smallest differences between the wastewater consumption measured and the prescription-based consumption measured. Additionally, a study out of northeast China found that out of 11 antibiotics across five classes, azithromycin was detected in 100% of urban wastewaters sampled during a 10-month period. Azithromycin was also shown to have the highest concentration (median = 276 ng/L) in sampled wastewaters. The highest distribution of ARGs was observed in hospital wastewater which supports the claim that antibiotics provide selective pressure for the formation and transmission of resistance genes [30].

### 3.4. Community vs. Hospital Wastewater

A notable comparison studied in the current literature is the difference between community wastewater influent and hospital wastewater discharge/effluent within the population sampled. Amongst the studies reporting this comparison (*n* = 7; 24%), hospital effluent appeared to exhibit greater abundance of clinically relevant resistance genes and pathogens compared to the wastewater influent [6,13,16,29,31], reflecting the selective pressure exerted by medical antibiotic usage. For example, a study out of China showed that there was a significantly positive correlation (*p* < 0.05) among antibiotics, ARGs, *intl1*, and 16S rRNA [31]. Comparatively, community influent may contain a broader array of environmental ARGs given the multiple sources of input, such as households, industry, and agriculture [7].

Over a 13-month monitoring period, one hospital measured higher antibiotic agents and metabolites compared to community influent, suggesting hospitals could be considered high risk facilities for contributing to AMR within communities [6]. ARGs detected from hospital effluent were also detected in raw urban wastewaters; however, at varying frequencies [32]. It has also been shown that the microbial community composition in wastewater differs between hospitals within the same city at both the phyla and genera levels [13]. This may be due to the difference in specialties as well as patient demographics (i.e., age) at each hospital. ARG profiles of these hospital wastewaters showed a higher prevalence and variety of ARGs in the phage fraction overall. However, another study demonstrated that *mcr-1/3/4/5*, *sul4*, and *gar* genes were detected in both municipal and hospital wastewater at relatively similar abundances [33]. This may support the assertion that certain ARGs may now regularly persist in the human gut microbiota in discrete populations [34]. This could have significant epidemiological implications, namely the ineffectiveness of antibiotics as a treatment option for bacterial infections.

The increased number of pharmaceutical ingestion and nosocomial infections in hospitals promotes the ideal environment for inter- and intraspecies transfer of ARGs [31]. During the COVID-19 pandemic, hospitals were leveraged in several wastewater-based epidemiology applications to detect and monitor SARS-CoV-2 as well as AMR. In one report, three hospitals with and one hospital without COVID-19 patients were compared where interestingly, notable overlap of several species of pathogenic bacteria was observed including Gram-negative species such as *Escherichia coli*, *Morganella morganii*, *Salmonella enterica*, and *Citrobacter fruendii* as well as Gram-positive species such as *Enterococcus faecalis*, *Enterococcus faecium*, and *Clostridium perfringens* [12]. Wastewater collected from these four different hospitals was found to have 61 out of 87 ARGs understood to confer resistance to major antibiotic classes including beta-lactams, fluoroquinolones, and aminoglycosides which showed the highest resistance rates. Noted as an emerging nosocomial infection, *Aeromonas* spp. is another ARB that was reported in the literature reviewed. The percentage of resistant *Aeromonas* spp. was significantly higher in hospital than in communal wastewater (*p* < 0.001), and co-trimoxazole and ciprofloxacin resistance in *Aeromonas* spp. from nursing home wastewater was significantly higher (*p* < 0.001) than from communal wastewater [16]. Moreover, another study suggests that an increase in relative abundance of *Aeromonas* spp. could be linked to increased abundance of *mcr-3* and *mcr-5* genes [33].

### 3.5. Conflicting Reports of Temporal Variation and Seasonal Influence on AMR

Of the studies reviewed, 7 (24%) specifically investigated temporal variability. Some (*n* = 4; 14%) showed a seasonal effect on the presence of ARGs. A study reported clarithromycin and erythromycin loads in wastewater of 32.8 ± 8.7 g/day in winter and 14.5 ± 4.2 g/day in the summer [6] which may be due to higher prescribing in winter months when respiratory illnesses typically peak. When observing variation on a much smaller scale, it was observed that the abundance of six different classes of antibiotics associated with ARGs (aminoglycosides, sulfonamides, quinolones, beta-lactams, macrolides, tetracyclines) were greater (*p* < 0.05) on weekends than weekdays [31], suggesting that the weekly fluctuation of patients may have an impact on the antibiotic profiles of untreated wastewater. Data from a 12-month study in southern California showed that many bacterial genera displayed a bimodal periodicity with higher transcript abundance during the winter and summer seasons [21].

Interestingly, some studies (*n* = 3; 10%) reported minimal or no seasonal effect on conferring AMR. There was no significant seasonal variation (*p* = 0.8210) observed in the total abundance of ARGs in activated sludge of two WWTPs when comparing April 2018 (NSWWTP = 0.27, FTWWTP = 0.24) to November 2018 (NSWWTP = 0.28, FTWWTP = 0.29) in the Guangdong province of China [19]. The most abundant ARGs identified in this study included genes associated with the antibiotic classes of multidrug resistance (27.78%), sulfonamide (20.03%), and bacitracin (15.43%). Furthermore, a study conducted in Iran demonstrated no clear seasonal effect as spring, summer, and autumn all had the same rate of abundance of ARGs with a slightly lower rate in winter with no significant (>0.05) correlation [35].

### 3.6. Understanding AMR Variability Through WBE

The use of wastewater-based epidemiology to assess the presence and conference of AMR within and between communities demonstrates significant potential to enhance current surveillance methods across the globe. However, several needs for understanding AMR variability through WBE persist in the current literature (Table 2). While several studies reported similarities in the types and frequencies of ARB, ARGs, and antibiotic residues detected and measured, there are also reports of variability that suggest the level of influence from the specific populations measured, such as context-specific drivers within each community (i.e., trends of pharmaceutical use, agricultural practices, industry, etc.), as well as standardization of methodology across studies reported. For example, when reviewing the reported data between hospitals and community wastewater, one explanation for the variation in data may be due to the number of hospitals that participated in the study. Most studies that analyzed hospital effluent (*n* = 9) only sampled from one hospital within a given community [6,7,8,25,30,31,32,33,36]. Increasing the number of hospital sites in these comparison studies will offer more robust conclusions regarding ARG presence, abundance, and influence as a function of hospital presence within communities [37].

One notable variable in the use of WBE is the sample collection approach. Hospitals and other medical centers, such as veterinary practices, serve as hotspots for pathogens, pharmaceuticals, and other microbial agents that may discharge into municipal and environmental wastewater streams [7]. Wastewater-informed data collected directly from these sites (i.e., near-source or building-level sample collection) can offer unique and preliminary information on potential disease outbreaks and how certain activities such as overuse of antibiotics may contribute to the overall resistome in local and downstream wastewater matrices. Of similar importance is the adoption of within-sewershed (i.e., neighborhood- or community-level) monitoring which can capture multiple sub-areas within a given larger municipality, city, or region and offer contextual and actionable data driven by WBE [38]. Prior to COVID-19 global pandemic, the predominant method for sample collection using WBE was largely focused on municipal wastewater treatment plants, which capture entire cities but may not provide an appropriate level of geospatial resolution needed to offer meaningful insights of population-level activity. This shifted during the pandemic in order to capture pertinent public health data between and across communities within a sewershed, offering groundbreaking evidence of the importance of such an approach and opened up the opportunity to explore its utility in other applications, such as AMR [38]. For example, a study conducted in eastern China showed that industrial parks have vastly different ARG profiles when compared to municipal WWTPs [14]. Industrial Park wastewater treatment plants treat substantial amounts of comprehensive wastewater derived from industrial production and human activity, potentially creating opportunities for increased resistance. Results from this multi-omic investigation revealed the dominant ARG subtypes were multidrug resistance genes (MDRGs) (*msbA*), the top five being *evgS*, *msbA*, *cpxA*, *MexW*, and *mtrA*, all of which were not reported in the other studies analyzed that only measured influent from WWTPs. These observations offer robust insights on the origination, development, dissemination, and risk assessment of industrial park wastewater streams as a significant source of resistance and warrants further study to conduct more within-sewershed monitoring, targeting these localized hotspot areas for future work in using WBE to track AMR [14].

Also of note is the issue of growing populations that place significant strain on the aging wastewater treatment infrastructure throughout the globe, and particularly in the United States [39]. It is becoming increasingly apparent that the removal efficiency of antibiotics and other pharmaceuticals during the wastewater treatment process is continuously challenged, further compounding an already complex problem, and raising the risk of downstream contamination of aquatic ecosystems post-treatment. However, WWTPs are uniquely positioned to support both the surveillance and management of the global antimicrobial resistance crisis. Using WBE to survey antimicrobial resistance within communities, including continuous trends of antibiotic usage and associated ARGs and ARB, may act as a complementary approach for current conditions and as an early warning system to detect resistance patterns and respond accordingly [40].

### 3.7. Additional Considerations for WBE-AMR: The Role of Biological Mechanisms

Underlying mechanisms that contribute to AMR are also important to consider. For example, horizontal gene transfer (HGT) plays a significant role in the dissemination of ARGs in wastewater microbial communities. Studies suggest that multi-resistant bacteria acquire ARGs through HGT during the wastewater treatment process [15]. HGT has been shown to take place in the activated sludge of a WWTP where there is a large accumulation of biomass containing a diverse array of microorganisms [19]. This method of information distribution is detrimental to controlling ARGs because it allows for the rapid dissemination of resistance within microbial communities. Additionally, activated sludge systems serve as environments where diverse microbial populations exist in concentrated quantities which facilitate the transmission of ARGs among pathogens and may compromise the treatment process. Many multi-resistance genomic determinants (MRGDs) containing certain ARGs are potentially associated with plasmids and could be transmitted via HGT in the presence of antibiotic-mediated selection. These plasmids not only can persist but may also evolve within environmental microbial communities [23]. Residues of antibiotics that enter the treatment system from populations accelerate the generation of ARGs and the horizontal transfer of genes, only further aggravating an already complex issue when faced with mitigating resistance development within the wastewater treatment and management infrastructure.

Mobile genetic elements (MGEs), such as plasmids and phages, are a form of genetic material that can move within a genome and from one species to another [41]. One study observed that resistance genes to three antibiotics (carbapenems, sulfonamides, and tetracyclines) along with MGEs were found at a high relative abundance (>$10^{-4}$ gene copies/16S rRNA gene copies) in hospital wastewater, suggesting that elevated amounts of antibiotic residues in hospital wastewater provide an enriching environment for ARGs [36]. Another study showcased the role of phages serving as reservoirs of ARGs, noting that strategies to prevent and eliminate ARGs should also include phages [32]. By understanding how and when genetic information is transmitted between species, researchers are better equipped to track and predict future spread of resistance.

Despite WBE having the potential for substantial and informative public health monitoring, current WBE practices still exhibit limitations that restrict its widespread application. One major challenge is the lack of standardized sampling and analytical methods, which makes it difficult to compare results across studies and regions [42]. The complexity of wastewater matrices can interfere with the detection and quantification of biological and chemical targets [43]. WBE often struggles to link findings to specific populations due to the pooled and anonymized nature of sewage. Additionally, socioeconomic factors such play a vital role so much that lower income nations are faced with not only the burden of disease but also poor infrastructure that contributes to improper sanitation [44]. Together, these challenges highlight the need for improved methodological consistency and robust validation before WBE can reliably support routine public health surveillance.

### 3.8. Research Gaps and Recommendations for Future Work

While there was some overlap, the variability observed between studies as a result from this review confirms that future work is needed to determine a standardized approach to leverage the use of WBE to combat antimicrobial resistance across the globe. A common limitation of the papers analyzed is the length of the data collection period. Most studies collected a limited number of samples (<30) over a short period of time (≤6 months). Longitudinal studies are necessary to assess temporal and seasonal variability in the abundance and diversity of ARGs in wastewater and their potential implications for public health. Additionally, considering other factors such as precipitation events and how that influences ARG abundance could strengthen data insights [45]. Measurements of other population-related drivers of AMR, such as heavy metals, non-antibiotic pharmaceuticals, and other contaminants such as microplastics, also require further investigation to understand co-resistance mechanisms within community wastewater streams that are heavily influenced by anthropogenic factors [8].

Improvements in wastewater treatment technologies that are comparable to continuous growing populations may aid in the reduction in ARG load entering back into the environment and eventually cycling back into the community [46]. Improving existing initiatives for antimicrobial stewardship practices to promote the responsible use of antibiotics in healthcare and agriculture may also reduce the overall load and burden on WWTPs. Evaluating the effectiveness of these practices in a variety of contexts is necessary for their broader adoption and can effectively be accomplished using WBE given its inherent near real-time benefits to support intervention efficacy assessment. WBE programs that not only monitor antibiotic resistance trends via ARB, ARGs, and antibiotic residues but also consider the underlying mechanisms and co-contaminants that further complicate the ability to monitor resistance within communities is strongly encouraged (e.g., MGEs, HGT, microplastics, metals, etc.). Investigating how these elements interact with various bacterial populations in wastewater may reveal critical insights into the persistence and dissemination of resistance traits that would ultimately inform relevant public health strategies.

The threat of AMR continues to increase as bacteria evolve to resist the effects of antibiotics, making infections harder to treat, increasing healthcare costs, and costing millions of lives each year [1]. WBE offers a unique tool for tracking AMR in communities by detecting and quantifying antimicrobial-resistant genes in wastewater, providing a real-time snapshot of local resistance patterns. This approach can be crucial for early detection, mapping resistance hotspots, and informing targeted interventions. However, leveraging WBE effectively to combat AMR requires a multi-faceted approach, with many stakeholders involved in order to be successful. To ensure that WBE reaches its full potential, policy development plays a pivotal role in setting clear guidelines for data collection, analysis, and sharing across regions. Governments must create frameworks that support the integration of WBE data into existing public health systems, ensuring timely responses to emerging threats.

**Figure 3:**
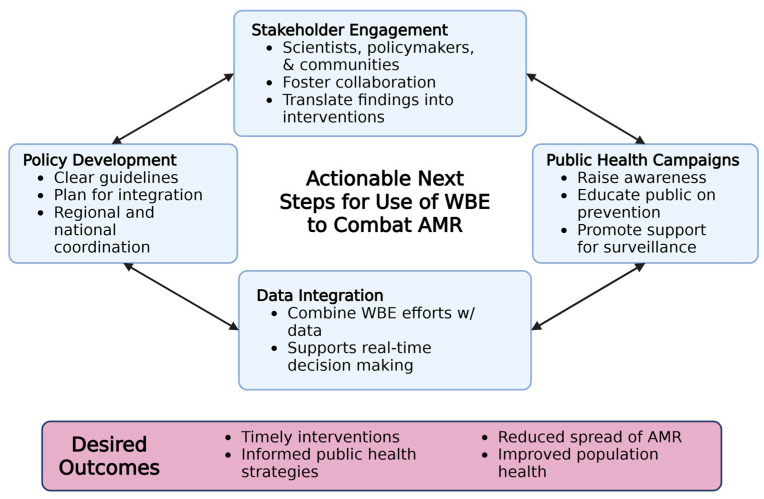
Framework for future wastewater-based epidemiology initiatives to combat antimicrobial resistance.

Public health campaigns are also essential, as they can increase awareness about the dangers of AMR and the role of wastewater monitoring in early detection. These campaigns can encourage appropriate antibiotic use and raise public support for investments in wastewater surveillance infrastructure while also taking geographical and socio-economic biases into consideration. Additionally, stakeholder engagement is key for driving collaboration between scientists, policymakers, healthcare professionals, and communities. Together, these groups can ensure that WBE findings are actionable, influencing national strategies and local interventions. Data integration is another critical element; by combining WBE data with traditional epidemiological surveillance, environmental monitoring, and clinical data, public health authorities can develop more accurate and comprehensive AMR mitigation strategies. This integration allows for real-time updates on AMR patterns, providing the scientific community with actionable insights to curb the rise in antimicrobial resistance effectively. This framework with actionable suggestions for future investigation via WBE are summarized in Figure 3.

## 4. Conclusions

The use of wastewater-based epidemiology to monitor antibiotic resistance remains a relatively underexplored application compared to others (i.e., pathogen surveillance, substance use) despite its far-reaching implications for protecting human health and environmental sustainability. This is likely due to the complex and multifaceted nature of AMR and the multitude of influences thereof. Addressing this requires a comprehensive understanding of the sources, fate, and consequences of antibiotic resistance in wastewater, as well as concerted efforts to develop and implement effective mitigation measures. The current literature highlights the urgent need for comprehensive surveillance and intervention strategies to curb the spread of antibiotic resistance within communities. Future research should focus on elucidating the impact of emerging contaminants such as heavy metals and microplastics on ARG dissemination, as well as implementing targeted efforts to assess the efficacy of advanced treatment technologies in the reduction in ARGs and mitigation of environmental and human health risk. Continued research and global collaboration within the scientific community in tandem with policymakers and stakeholders is essential to safeguard public health and preserve the efficacy of antibiotics and other antimicrobial agents in the face of evolving resistance threats.

## Figures and Tables

**Table 1:** Overall summary of reported methods, results, and needs for future work obtained from articles analyzed (see Table S1 for comprehensive information).

Sample Collection	Identification Methods	Results	Future Work
Average number of samples collected: 47 * Average number of locations: 5 * >50% reported use of 24-hr composite samples Average length of study duration: 9 * months	Conventional PCR qPCR 16S rDNA sequencing LC-MS/MS Bacterial cultures Bioinformatics/metagenomics Antimicrobial susceptibility testing	Top genus of bacteria identified: *Escherichia*, *Pseudomonas*, and *Enterococcus* Top antibiotic classes identified: macrolides, beta-lactams, tetracyclines, fluoroquinolones, and bacitracin Top antibiotic resistance genes: *bla* genes, *sul1/2*, *ermB/F*, *intl1*, *qnrS*, *mcr*/*mbr*, and *tetM* Data for machine learning models Conclusions about environmental and human health impacts	Identify additional influential factors in antibiotic resistance propagation (i.e., HGT, environmental pollutants) Examine the role of phages in spreading ARGs Investigate the influence of seasonality and One Health implications Integrate findings into wastewater surveillance programs

* Rounded to the nearest whole number.

**Table 2:** Needs to understand AMR variability using WBE.

Context-Specific Drivers	Sample Collection Approaches	Future Challenges
Pharmaceutical use Agricultural practices Industry run-off Anthropogenic contaminants (heavy metals, personal care products, etc.)	Facilities sampled (hospitals, WWTPs, industrial plants, etc.) Frequency of sampling Collection methods (composite samplers, grab samples, etc.)	Growing populations Aging infrastructure Removal efficiency of WWTPs Standardized surveillance and management protocols

## Data Availability

The original contributions presented in this study are included in the article/Supplementary Material. Further inquiries can be directed to the corresponding author.

## Supplementary Materials

The following supporting information can be downloaded at https://www.mdpi.com/article/10.3390/microorganisms13092073/s1, Table S1: Comprehensive summary of articles analyzed focusing on the study objectives, methods, major findings, and reported limitations of the work.

## Abbreviations

The following abbreviations are used in this manuscript:

ARG	Antimicrobial resistance gene(s)
AMR	Antimicrobial resistance
ARB	Antibiotic-resistant bacteria
WBE	Wastewater-based epidemiology
WWTP	Wastewater treatment plant
HGT	Horizontal gene transfer
MGE	Mobile genetic elements
AA	Antibiotic agents (i.e., antibiotics, antimicrobials)
