# Supplementary material for: Use of Wastewater to Monitor Antimicrobial Resistance Trends in Communities and Implications for Wastewater-Based Epidemiology: A Review of the Recent Literature
Source: Microorganisms. 2025 Sep 5;13(9):2073. doi: 10.3390/microorganisms13092073 (PMC12473023; doi:10.3390/microorganisms13092073)
Supplement: Supplementary file 1 [file microorganisms-13-02073-s001.zip › microorganisms-3748871-supplementary.pdf]

Table S1. Comprehensive summary of articles analyzed focusing on the study objectives, methods, major findings, and reported limitations of the work.

Source	Objectives	Collection Methods	Findings	Limitations (if stated)
**Sims et al., 2023**	• To test correlations between antimicrobial agents (AAs) and corresponding ARGs<br>• Provide better understanding of AA-ARG associations, understand the role of wastewater in the dissemination of AMR<br>• Understand AA and ARG contributions in hospital vs. community wastewater	• Sampling occurred over a 13-month longitudinal period<br>• 24-hr composite samplers<br>• WWTP influent and hospital effluent sampled	• Several AAs had higher load in winter than summer<br>• Abundance of microorganisms is proportional to size of human population<br>• Hospitals showed higher AAs and metabolites<br>• Hospitals could be high risk for AMR	• Limited selection of both AA and ARG targets<br>• No account for chemical drivers of AMR (i.e., biocides, heavy metals)<br>• Lack of monitoring in rural areas to investigate geographically driven AMR drivers<br>• No account for veterinary usage
**Zhao et al., 2022**	• To investigate the ARGs, bacteria and viruses in the influent and effluent of six decentralized sewage treatment facilities	• Sampling occurred over the course of one month<br>• Influent and effluent were sampled from facilities that receive both	• Bacitracin-ARGs were most predominant in rural wastewater<br>• ARGs were identified in both bacterial and viral community<br>• Some ARGs were correlated with bacteria and phages	

		livestock and household wastewater		
**Pires et al., 2023**	• To evaluate the presence of ARGs in the phage fraction of hospital and raw urban wastewaters<br>• The potential role played by hospital wastewaters on the dissemination of ARGs in the phage fraction	• Samples collected over four months<br>• 24-hr composite samplers<br>• WWTP influent and hospital effluent sampled	• The difference between [ARG] in hospital wastewaters was not statistically significant neither was the frequency profile of ARGs<br>• ARGs detected in hospitals were also detected in raw urban wastewater but at different frequency profiles<br>• Results underline the potential for ARG transmission by phages	
**Kang et al., 2022**	• To monitor ARG resistome and culturable MRB in the presence of 8 different antibiotics	• Samples collected over five months<br>• Influent, raw sludge, activated sludge, mixed sludge, and digested sludge sampled from a WWTP	• Findings suggest that MRB acquire ARGs through HGT during the WW treatment process<br>• There was a trend of the occurrence of MRB through HGT of ARGs and the prevalence of transposon-mediated ARGs in sludges of	

			WWTPs during two seasonal periods	
**Tian et al., 2022**	• To determine long-term dynamic profiles of ARGs, identify core ARGs, and expose the dynamic variations of ARGs in AS after wastewater treatment process upgrades	• Two WWTPs sampled<br>• Sampling occurred over 32 months for NSWWTP and 19 months for FTWWTP	• No significant seasonal variation was shown in ARG profile<br>• Treatment process upgrades could curb transmission of ARGs	
**Hutinel et al., 2022**	• To evaluate the presence and potential spread of a set of mobile ARGs of emerging concern	• 44 samples collected over a five-year period<br>• 24-hr composite samplers<br>• One WWTP and one hospital	• Results indicate wide dissemination in a country where certain genes have rarely or never been detected in a clinical setting<br>• ARGs could be present in the gut flora of contributing populations	
**Guo et al., 2021**	• To identify the bacterial community composition and prevalence of ARGs in the wastewater from three hospitals<br>• Determine special interventions for protection against infections and pre-treatment of	• Three hospitals sampled<br>• Samples collected over three non-consecutive days during one month	• Community composition of bacteria in WW was different between hospitals at both phyla and genera levels with significant different communities of bacteria within them<br>• The same ARGs contributed to	• There was no physiochemical analysis for raw wastewater which may be affected by the seasons or other environmental conditions

	wastewater before discharge		different bacterial genera with various relatives in WW from the groups	
**Zare et al., 2021**	• To assess three important ARGs conferring resistance to sulfonamides in both bacteria and DNA fractions of wastewater samples	• Three wastewater locations sampled: raw sewage, before treatment, and after treatment<br>• Sampling occurred over a 12-month period with three samples collected per season	• The presence of ARGs found in bacterial DNA was higher than phages<br>• No clear seasonal effect<br>• Results defend thought that phages may be carrier for HGT of ARGs in environment	
**Liu et al., 2021**	• To investigate the occurrence and abundance of ARGs and MGEs, the structure of microbial communities, and concentration of antibiotics and other relevant environmental factors in wastewater	• Sampling occurred over one month<br>• Five sites sampled within an ornamental fish market	• Water environmental factors play important co-selection roles on ARG distribution<br>• Selection roles of antibiotics coupled with water environmental factors and their induced shifts in the microbial communities are the determining drivers for the existence and prevalence of ARGs	
**Asghari et al., 2021**	• To characterize the microbial populations in	• Untreated wastewater samples were	• High prevalence of *P. aeruginosa* and *E. coli*	

	hospital wastewater and investigate the prevalence of beta-lactamase resistance genes	collected from a hospital<br>• Sampling occurred twice during a 12-month period<br>• 24-hour composite samplers	carrying ARGs in hospital wastewater<br>• Highest resistance rates found for sulfamethoxazole<br>• Results indicated increase in resistance of identified bacteria to several antibiotics	
**Rothman et al., 2022**	• To investigate the transcriptomic diversity of microorganisms in wastewater<br>• To assess what AMR genes are actively being transcribed in WW<br>• To determine if there are conserved biochemical pathways across wastewater	• Eight WWTPs were sampled<br>• Collected a total of 275 1-liter 24-hour composite influent samples across the WWTPs<br>• Sampling occurred within a 12-month period	• WW metabolism is largely consistent across WWTPs over time<br>• Top human pathogens identified include *K. pneumoniae*, *A. baumannii*, *P. aeruginosa*, and *Enterobacter* spp.<br>• Many genera displayed a bimodal periodicity	• Methodology may have resulted in nucleic acid degradation and therefore affected results<br>• Results may be confounded by sequencing difficulties
**Wen et al., 2023**	• To investigate changes of fecal coliform bacteria (FCB) concentration along with processes in WWTPs<br>• Determine characteristics and	• Samples were collected during three non-consecutive months within a 12-month period<br>• Samples were collected during three consecutive days	• FCB is more effectively limited by UV radiation during summer<br>• ARGs involved in resistance phenotype were not clear<br>• Significant amount of FCBs retained in sludge form of	

	changes of ABR of FCB community<br>• Clarify how seasonal changes impacts composition and ABR of FCB community	• Sampling points consisted of influent, primary and secondary sedimentation tanks, and effluent	dehydrated solids rather than being inactivated through biological processes<br>• *Klebsiella* spp. has the highest resistant rate in FCB	
**Czatzkowska et al., 2023**	• To evaluate the influence of combined treatment of landfill leachate (LL)<br>• Municipal wastewater on the spread of AR based on analyses of physiochemical parameters, microbial diversity, and abundance of selected ARGs	• Two WWTPs were sampled<br>• LL, untreated wastewater, and treated wastewater discharged into a nearby river were all sampled<br>• 24-hr composite samplers<br>• Samples were collected three times in three seasons (spring, summer, autumn) during one calendar year	• Combining treatment of LL and wastewater may result in increased pollution of river water receiving effluents<br>• Exposure to LL induced changes in microbial abundance and the interactions between microbial taxa in wastewater<br>• mexF gene identified as potential marker of LL	• Results do not validate the conclusion that the co-treatment of landfill leachate with municipal wastewater contributes to increased ARG load in the receiving river
**Johar et al., 2023**	• To determine bacterial diversity and identify ARG profiles in hospital wastewater pathogens obtained from COVID-19	• Five hospitals were sampled<br>• Sampling occurred over a seven-month period	• Observed 27 different bacterial species in the samples<br>• 61 ARGs were detected in total<br>• Highest number of ARGs was observed	

	isolation hospitals compared with non-COVID-19 facilities during the pandemic	• Three samples were collected from each site on different days	for the COVID-19 patient site and the lowest number of ARGs was found at the non-patient site	
**Verburg et al., 2019**	• To investigate the AMRB contribution of clinical and non-clinical wastewater sources to the wastewater chain and their dependence on the antibiotic usage, the fate of the AMRB in the treatment plant, and the impact of WWTP discharges in terms of AMR on receiving surface water	• Five sampling locations<br>• 24-hr sampling<br>• In addition to the five locations, surface water samples were collected from the receiving surface water<br>• Samples were collected during a 12-month period, every four weeks, two days in a row	• The highest percentage of resistant bacteria was found in hospital WW<br>• The contribution of hospital WW to AMR in influent was only 1% of total WW entering WWTP<br>• *Klebsiella* spp. and *Aeromonas* spp. showed larger differences in resistance percentages when compared to *E. coli*	
**Su et al., 2023**	• To investigate the transfer, elimination, and accumulation of ARGs undergoing decentralized household WWTPs<br>• Compare the effect of intervals for discharge of excess sludge on the fate of ARGs	• 62 samples were collected over a two-month period<br>• Samples were collected from seven sites along the treatment process including influent, biological treatment, sedimentation,	• Four ARGs and one MGE was eliminated by sedimentation and desludging in WWTP storage tank<br>• Genes in SS with small size remained in WW post-treatment<br>• Pre- and post-aeration was found to promote ARG transfer	

	• Investigate the existing states of ARGs	post-aeration, and re-sedimentation<br>• Sludge was sampled in three locations		
**Wang et al., 2023**	• To investigate the changes in ARGs, MGEs, and bacterial communities in wastewater from different treatment processes<br>• Identify the main factors driving the spread of ARGs, and find co-occurrence of ARGs and host bacteria, including potential pathogens in WW	• Three WWTPs were sampled<br>• Samples included influent, main process effluent, and final effluent<br>• Samples were collected three times	• Disinfection, particularly chlorination, showed no significant effect on removing ARGs<br>• WW quality index, MGEs, and insertion sequences were the main factors affecting ARG abundance	
**Quintela-Baluja et al., 2019**	• To clarify which ecosystems and in-process mechanisms most strongly impact ARGs found in downstream receiving waters to develop better-informed WWTP mitigation solutions for reducing AR releases to the natural environment	• Sampling was performed during a consecutive three-month period<br>• Sampling from two hospitals and one municipal WWTP<br>• Samples include influent, effluent, recycled activated sludge, water	• Source wastewater ARGs may be more important to WWTP effluents than believed<br>• Greater ARG richness and higher levels of ARGs/genome prevail in hospital sources might disproportionately influence ARGs entering the WWTP	

		column and sediment from upstream and downstream of the WWTP discharge point		
**Shen et al., 2022**	• To explore removal capacity of WWTP on antibiotics, ARDs, and bacteria in summer and eval impact of effluent from WWTP on receiving water<br>• Compare data of antibiotics, ARDs, and microbial diversity in WWTP and receiving waters in summer and winter, analyze differences, and explore significance<br>• Examine difference between antibiotics, ARDs<br>• Bacteria in WWTP and receiving waters to clarify connection network	• Sampled one WWTP and 2 rivers<br>• Samples include upstream river, mixed river, effluent, downstream river, upstream estuary, and downstream estuary	• Removal capacity of bacteria and antibiotics in summer was weaker than that in winter<br>• The relationship between bacteria, antibiotics and ARDs is deeply affected by the season	• The recovery rate of antibiotics was only evaluated in the influent water of WWTP<br>• Method only consisted of single sampling so the study's results cannot describe a stable effect of WWTP in removing antibiotics
**Lee et al., 2021**	• To assess impact of stormwater-related disturbance and	• Samples collected from a river that receives	• WWTPs are potentially important intervention points for	

	resilience of the resistome of the Murg River by monitoring temporal dynamics of the resistome during stormwater events that lead to combined-sewage bypass, identify key source(s) which contribute most to increase of riverine resistance level<br>• Assess the impact of stormwater events in terms of multi-resistance risk factors	wastewater effluent<br>• Event-based sampling over a consecutive three-month period	preventing/minimizing discharges of bypass-borne ARGs<br>• Temporal persistence of bypass-borne ARGs in receiving water should be considered when interpreting fate of aquatic ARGs during/after stormwater events<br>• Risk of exposure to multi-resistance risk factors increased profoundly in the bypass-receiving river due to bypass inputs during stormwater events	
**Kumar et al., 2020**	• To determine the prevalence of ARB, ARG, and MDR in wastewaters in Sri Lanka and compared to those produced in a city in India<br>• Statistically trace the imprints of treatment by comparing ARB, ARG, and metals in	• Five WWTPs and one hospital sampled	• *E. coli* prevalence decreased during treatment but remaining bacteria could adapt in presence of Aas<br>• WWTPs showed higher rates of AMR and consistent increase in AMR post-treatment<br>• Metal concentrations found within permissible limit but	

	the influent and effluents analyzed		likely to influence ARBs	
**Osunmakinde et al., 2019**	• To investigate the abundance and diversity of the bacterial communities in influent and effluent wastewater samples from three WWTPs in South Africa	• Three WWTPs were sampled<br>• Samples included influent and effluent	• *Pseudomonas* spp., *Bacillus* spp., *Streptococcus* spp., and *Staphylococcus* spp. were among the 17 genera that dominated all influent and effluent samples<br>• Pathogens can be dispersed via WWTPs	• 16S RNA approach requires amplification which may introduce some bias and therefore affect the accuracy of the whole picture of bacterial communities actually present in the sample
**Cai et al., 2021**	• To evaluate the temporal variations and pollution profiles of antibiotics, ARGs, *intI1*, and 16S rRNA genes released from inpatient dept WW intraday and intraweek, the removal pattern through self-contained WWTP of this hospital, the relationship between the	• Samples collected from one hospital<br>• Two sampling campaigns performed (intraday and intraweek) in triplicate<br>• Sampling occurred during one week	• The significantly positive correlation among antibiotics, ARGs, *intI1*, and 16s rRNA implies risk of selective pressure HGT, and vertical propagation of ARGs in hospital effluent<br>• Significant impact of the daily schedule of inpatients and the treatment process on occurrence profiles of antibiotics and ARGs in HWW	

	occurrence of ARGs and the presence of antibiotics in HWW, and the effects on the occurrence regularity by the daily schedule of inpatients			
**Wu et al., 2023**	• To investigate the occurrence and distribution of ARGs, ARGs host and pathogens along the wastewater biological treatment process, the main functional sections of one typical large-scale industrial park WWTP to determine the removal efficiency of ARGs and pathogens using metagenomics analysis<br>• Asses the environmental risk of the associated ARGs through a metagenome-based ARG ranker model	• Samples collected from one industrial park WWTP<br>• Triplicate samples collected from influent, anaerobic tank, secondary sedimentation tank, and refluxed activated sludge	• For ARGs, MDRGs were the dominant ARGs<br>• ARG abundance and their health risk are not correlated	

**Teixeira et al., 2023**	• To establish a set of genes that could be used as representative biomarkers of antibiotic resistance contamination for monitoring wastewater and downstream aquatic environments	• 41 samples from five countries sampled<br>• Collection sites included airport, hospital and municipal urban WWTPs<br>• Sampled influent, effluent, and tertiary effluent	• The use of proper biomarkers may be a cost-effective method for monitoring wastewater<br>• When other biomarkers are below the limit of detection, *intI1* may still be used to indicate the presence of low-abundance ARGs	
**Sun et al., 2023**	• To identify the distribution of ARGs and antibiotics from various urban wastewater and clarify their pollution characteristics in different urban wastewater<br>• Investigate the co-occurrence patterns between ARGs and antibiotics	• Sampling occurred over a ten-month period<br>• Samples included community sewage, livestock, hospital, pharmaceutical factory, and WWTP influent (domestic and industrial wastewater)<br>• Samples were taken when there was no rain for three consecutive days	• The emergence of contaminants in sewage water, such as ARGs and high pathogen levels, poses a potential risk to public health and the aquatic ecosystem	
**Liu, B. et al., 2023**	• To quantify and analyze pharmaceuticals for	• Four WWTPs were sampled	• There was a significant difference in the distribution of	

	their correlation with typical ARGs as well as *intl1* in wastewater and sludge to build better prediction models	• Samples include influent, effluent, and sludge	pharmaceuticals between sewage and sludge<br>• Most pharmaceuticals showed positive correlation with ARGs in the sewage but negative correlation with ARGs in the sludge	
**Escola Casas et al., 2021**	• To develop a more powerful WBE approach that allows reliable back-calculation of a wide variety of pharmaceuticals from different classes by using highly spatially and temporally specific prescription data as well as accounting for wastewater levels of pharmaceuticals that result from both urinary and fecal excretion	• 24-hr composite samplers<br>• Sampling occurred over five consecutive days<br>• Samples were collected from two cities of different sizes in the same region	• Antibiotics showed the smallest difference between MC and PC when compared to other pharmaceuticals	• Only public prescription numbers were available<br>• Only prescriptions for oral administration were counted<br>• OTC was not accounted in the prescription counts<br>• The number of defined daily doses (DDD) per prescription may differ from the total DDD of one whole treatment<br>• Excretion rates of

				pharmaceuticals reported in the literature is scarce and not up-to-date
**Beltran de Heredia et al., 2022**	• To reveal the potential spatio-seasonal patterns of the impact of WWTP effluents on AR in river sediments	• Three sampling points were selected in three river basins (upstream, effluent, and downstream from the WWTP)<br>• Two sampling campaigns were performed each for one month ten months apart<br>• Composite surface sediment samples were collected in triplicate	• Significant variations between high-water and low-water periods<br>• Positive correlations between ARGs and MGE relative gene abundances point out to the role of HGT in AR spread<br>• *sul1*, *intl1*, and *tnpA* show potential as genetic markers for AR risk	
